# Parenting Practices and Adolescent Risk Behavior: Rules on Smoking and Drinking Also Predict Cannabis Use and Early Sexual Debut

**DOI:** 10.1007/s11121-012-0286-1

**Published:** 2012-09-08

**Authors:** Margaretha de Looze, Regina van den Eijnden, Jacqueline Verdurmen, Evelien Vermeulen-Smit, Ingrid Schulten, Wilma Vollebergh, Tom ter Bogt

**Affiliations:** 1 Department of Child and Adolescent Studies, Faculty of Social and Behavioural Sciences, Utrecht University, PO Box 80.140, 3508 TC Utrecht, The Netherlands; 2 Netherlands Institute of Mental Health and Addiction, PO Box 725, 3500 AS Utrecht, The Netherlands

**Keywords:** Adolescents, Tobacco use, Alcohol use, Cannabis use, Early sexual debut, Parenting practices, The Netherlands, HBSC

## Abstract

Previous research has provided considerable support for idea that increased parental support and control are strong determinants of lower prevalence levels of adolescent risk behavior. Much less is known on the association between specific parenting practices, such as concrete rules with respect to smoking and drinking and adolescent risk behavior. The present paper examined whether such concrete parental rules (1) have an effect on the targeted behaviors and (2) predict other, frequently co-occurring, risk behaviors (i.e., cannabis use and early sexual intercourse). These hypotheses were tested in a nationally representative sample of 12- to 16-year-old adolescents in the Netherlands. We found that both types of rules were associated with a lower prevalence of the targeted behaviors (i.e., smoking and drinking). In addition, independent of adolescent smoking and drinking behaviors, parental rules on smoking predicted a lower prevalence of cannabis use and early sexual intercourse, and parental rules on alcohol use also predicted a lower prevalence of early sexual intercourse. This study showed that concrete parental rule setting is more strongly related to lower levels of risk behaviors in adolescents compared to the more general parenting practices (i.e., support and control). Additionally, the effects of such rules do not only apply to the targeted behavior but extend to related behaviors as well. These findings are relevant to the public health domain and suggest that a single intervention program that addresses a limited number of concrete parenting practices, in combination with traditional support and control practices, may be effective in reducing risk behaviors in adolescence.

## Introduction

In many Western countries, adolescent risk behaviors are among the top priorities in the public health domain. Specifically, numerous prevention and intervention programs have been developed to reduce young people’s engagement in smoking tobacco, drinking alcohol, using cannabis, and early sexual intercourse. In recent years, researchers have observed two trends in the development of such programs. First, public health programs aiming to reduce adolescent risk behaviors have increasingly focused on the role of parents (e.g., Koning et al. 2009, 2011a; Sigfúsdóttir et al. 2009). While, prior to 2004, most programs targeted adolescents directly, parents have recently been acknowledged more often as effective agents to affect young people’s behaviors. Second, there is a trend toward developing prevention practices that target multiple risk behaviors simultaneously, rather than targeting behaviors individually. Such broad programs may be beneficial for three reasons. First, research has shown that risk behaviors cluster together (Willoughby et al. 2004), which increases the likelihood of youth who engage in different risk behaviors simultaneously. Second, from an economic perspective, the implementation of broad programs that target multiple risk behaviors is more cost effective compared to the implementation of separate risk behavior-specific programs. Third, a single program that focuses on multiple risk behaviors, simultaneously, requires less effort from parents and increases their willingness to participate compared to implementing a variety of programs that each focus on a single behavior.

The present study aimed to provide insight into the association between parenting practices and a variety of risk behaviors that cluster together during adolescence. We thereby specifically focused on the following behaviors: smoking tobacco, drinking alcohol, using cannabis and having sexual intercourse. These four behaviors frequently co-occur during adolescence (Willoughby et al. 2004). Their interrelatedness may be explained by the fact that they are all adult-like behaviors that become increasingly attractive to young people as they grow up, while society does not (yet) accept young people’s engagement in these behaviors (Moffitt 1993, 2006). The role of parents to guide their children through this phase, by providing adequate parenting practices, is tested in this study. We examined to what extent general parenting practices (i.e., providing parental support and control) and specific parenting practices (i.e., setting concrete rules) predicted adolescent engagement in these behaviors. Furthermore, we examined whether concrete parental rules on smoking and drinking did not only predict adolescent smoking and drinking behaviors, but also engagement in cannabis use and early sexual intercourse.

## Parenting Practices and Adolescent Risk Behaviors

Previous research on parenting practices and adolescent risk behaviors can be divided into two categories: (1) studies that have focused on general parenting practices, most notably parental support and control (Baumrind 1966) and (2) studies that have focused on concrete parenting practices that target specific behaviors, such as parental rules on adolescent smoking and drinking (e.g., van der Vorst et al. 2007). With respect to general parenting practices, a combination of parental support and control (i.e., warm, caring parenting with appropriate supervision and control) is considered to contribute to the best mental health outcomes for young people. It has been related to lower degrees of adolescent alcohol use (Roche et al. 2008; Ryan et al. 2010), smoking (Castrucci and Gerlach 2006; Harakeh et al. 2010), cannabis use (Chen et al. 2005) and delay of sexual debut (de Graaf et al. 2010, 2011; Roche et al. 2008).

With respect to specific parenting practices, most research has focused on practices that target adolescent smoking and drinking. Among specific parenting practices aimed at reducing adolescent alcohol use, the strongest predictor is concrete parental rules on adolescent drinking behavior (van der Vorst et al. 2007). The association between concrete parental rules on smoking and adolescent smoking behavior has also been assessed; however, findings are mixed (Emory et al. 2010). While some studies found a strong association (Pennanen et al. 2012), other studies found a weak association (Andersen et al. 2004; Huver et al. 2006) or no association at all (den Exter Blokland et al. 2006; de Leeuw et al. 2010; Harakeh et al. 2005). The differences in outcomes are likely to be related to the use of different definitions of smoking rules. While some studies focused on house rules in general (Andersen et al. 2004; de Leeuw et al. 2010; Henriksen and Jackson 1998; den Exter Blokland et al. 2006; Harakeh et al. 2005), other studies focused on whether adolescents themselves were allowed to smoke at home (Huver et al. 2006; Pennanen et al. 2012). Anti-smoking rules specifically aimed at the adolescent appear to be more strongly related to adolescent smoking behavior compared to the more general house rules. To the knowledge of the authors, to date, no study has included a measure on whether adolescents are allowed to smoke by their parents at all, independent of the context (i.e., also outside their home, such as at a party with friends). Such rules can be expected to be even more relevant in predicting adolescent smoking behavior as they are directly aimed at the adolescent and are applicable to the adolescent's more general life.

While most previously conducted studies are based on cross-sectional or longitudinal data which do not necessarily include an intervention, and while the nature of parent-adolescent relationships is bidirectional (Keijsers et al. 2010), intervention studies suggest that family interventions focusing on increasing parental support, control, and rule setting are effective in reducing adolescent alcohol use (Smit et al. 2008) and tobacco smoking (Thomas et al. 2008), and that family interventions focusing on parental support and control are effective in reducing cannabis use (Bender et al. 2011; Soper et al. 2010) and delaying early sexual debut (Downing et al. 2011). These findings suggest that associations between parenting practices and adolescent risk behaviors at least partly reflect an effect of parenting practices on adolescent substance use and early sexual debut.

While previous studies related parental support and control to different types of adolescent risk behaviors, their relative effects, compared to concrete parental practices, are not clear. Moreover, concrete parental rules on smoking and drinking have only been related to the targeted behaviors, while their effects may extend to other risk behaviors due to high co-occurrence rates. Keeping in mind the recent developments of broad intervention programs involving parents, it would be relevant, from both a practical and conceptual point of view, to investigate to what extent general versus concrete parenting practices are important in predicting adolescent risk behaviors, and to determine whether concrete parental rules that target a specific risk behavior (e.g., smoking) also have an effect on other, related risk behaviors (e.g., cannabis use).

## The Present Study

In the present study, we examined these research questions based on a large, nationally representative sample of Dutch adolescents in secondary education. Consistent with existing literature, we hypothesized that parental support would be negatively associated with adolescent smoking, drinking, cannabis use, and early sexual debut. Furthermore, we expected parental control to be negatively associated with all four risk behaviors; however, this association would be mediated by concrete parental rules on smoking and drinking (as per existing precedents; e.g., van Zundert et al. 2006). Finally, we expected that concrete parental rules on smoking and drinking would not only be negatively related to smoking and drinking behaviors, but also to cannabis use and early sexual intercourse. To explore whether our results applied similarly to different subgroups of youth, we tested whether the associations found were similar for boys and girls, youth from different age groups (early vs. mid-adolescence), and youth with different educational levels (low vs. high). These analyses were relevant to determine the potential effectiveness of a broad intervention program for different subgroups of youth.

## Methods

The current sample was drawn from the Dutch Health Behaviour in School-aged Children (HBSC) survey. The HBSC study is a World Health Organization collaborative cross-national study on the health, health-related behaviors, and the social context of young people’s health. Consistent with the international study protocol (Griebler et al. 2010; Roberts et al. 2009), data from Dutch students in the first through fourth years of secondary education (12–16 year olds) were collected via an anonymous self-report questionnaire at secondary schools from October to November 2009. Schools were randomly selected from a governmental list of all secondary schools in the Netherlands after stratification based on urbanicity. In total, 68 schools participated in the study. Per school, three to five classes were randomly selected from a list of all classes in the first through fourth years. As the secondary education system consists of four educational levels that range from pre-vocational training to higher academic education, students from all educational levels were included, and the final sample was nationally representative in terms of the educational level of adolescents aged 12 to 16. Only students whose parents did not object to their child’s participation in the study were included in the study. The response rate within classes was 93 %, with the most important reason of nonparticipation in the study being illness.

The final sample included 5,642 students, who were representative of Dutch youth aged 12–16 years (*M* = 13.8; *SD* = 1.3) in the first 4 years of secondary education. 51 % of the respondents were boys, and 15 % had an ethnic minority background. With respect to educational level, 46 % of the respondents were classified as having a high educational level (i.e., they attended one of the two highest levels).

## Measures

### Adolescent Risk Behaviors

Risk behaviors included in our study were three different types of substance use and early sexual intercourse. We dichotomized smoking, binge drinking and cannabis use because the aim of our paper was to identify high-risk involvement in risk behaviors among adolescents.

#### Daily Tobacco Smoking

With respect to tobacco smoking, adolescents were asked: ‘How often do you smoke at present?’ The original answer categories (never, less than weekly, weekly but not daily, daily) were recoded into ‘no daily smoking’ and ‘daily smoking.’ As daily smoking is a crucial aspect of nicotine dependence (Jarvis 2004), daily smoking adolescents have an increased likelihood of smoking in the future and developing smoking-related health problems leading to premature deaths (Hublet et al. 2006).

#### Binge Drinking in the Previous Month

With respect to alcohol use, adolescents were asked: ‘How often have you, in the previous month, drunk five or more alcoholic drinks on one occasion (for example at a party or a night out)?’ Original answer categories (ranging from ‘never’ to ‘nine times or more’) were recoded into ‘never’ and ‘at least once.’ Regular binge drinking is considered an indicator of excessive alcohol use (as per Lammers et al. 2011) and has been related to a wide range of negative outcomes, including brain damage and neurocognitive deficits (Tapert et al. 2002; Zeigler et al. 2005), poor educational attainment (Hill et al. 2000), and adult alcohol dependence, illicit drug use, and psychiatric morbidity (Viner and Taylor 2007).

#### Lifetime Cannabis Use

Lifetime cannabis use was measured by the item ‘How often, in your entire life, have you smoked cannabis?’ The original answer categories (ranging from never to 40 times or more) were recoded into ‘never’ and ‘at least once.’ Cannabis use is rare among adolescents aged 12–16; if adolescents at this age already have experience with cannabis use, this is generally interpreted as an (extreme) risk behavior. Early cannabis use has been found to affect neurocognitive functioning (Schweinsburg et al. 2008) and has been associated with an increased risk for problems later in life, including substance abuse and dependence (Lynskey et al. 2003; Agrawal et al. 2004), depression (Patton et al. 2002), and psychosocial adjustment problems (Fergusson et al. 2002).

#### Early Sexual Intercourse

Students were asked whether they had ever had sexual intercourse. Answer categories were either ‘yes’ or ‘no.’‎ Early sexual intercourse was considered a risk behavior as it has been related to long-term negative sexual health outcomes, including increased sexual risk taking (de Graaf et al. 2012; Sandfort et al. 2008).

### Parenting Practices

The parenting variables in our model were included as latent constructs, which consisted of a number of categorical items as indicators. To describe the quality of these constructs, we will report the model fit of each latent construct separately in this section. Because the sample size was large and the chi square statistic is sensitive to sample size, we specifically focused on the Comparative Fix Index (CFI), Tucker-Lewis Index (TLI), and the Root Mean Square Error of Approximation (*RMSEA*) as indicators of model fit. The CFI and TLI are related to the total variance accounted for in the model; values greater than 0.90 are accepted and values greater than .95 are desired (Kline 2010). The *RMSEA* is related to the residual variance; values smaller than 0.10 are accepted and values smaller than .05 are desired (Kline 2010). For an acceptable model fit, at least two of the three indices need to be adhered to.

#### Parental Support

This construct was based on six indicators: (1) My parents show me that they admire me; (2) In my parents’ eyes, I do everything wrong; (3) My parents show me that they love me; (4) My parents often make me look ridiculous; (5) My parents support me in my activities; and (6) My parents treat me aggressively. Answer categories ranged from ‘definitely true’ to ‘definitely not true’ (Scholte et al. 2001). A confirmatory factor analysis revealed acceptable model fit statistics: *χ²*(9) = 728.59, *p* = .00, *CFI* = .96, TLI = .94, *RMSEA* = .12.

#### Parental Control

The construct parental control was based on three indicators: (1) Before you leave the house, do your parents want to know with whom or where you are going?; (2) Do you need your parents’ permission to go out at night?; and (3) If you go out at night, do your parents want to know afterward with whom or where you were? (adapted from Kerr and Stattin 2000). A confirmatory factor analysis revealed good model fit statistics: *χ²*(1) = 42.45, *p* = .00, *CFI* = .99, TLI = .97, *RMSEA* = .09.

#### Alcohol-Specific Rules

The construct alcohol-specific rules was based on four indicators: (1) I am allowed to drink one glass of alcohol when my father or mother is home, (2) I am allowed to drink several glasses of alcohol when my father or mother is home, (3) I am allowed to drink alcohol at a party with friends, and (4) I am allowed to drink alcohol on the weekends. Answer categories ranged from ‘definitely not true’ to ‘definitely true’ (adapted from Van der Vorst et al. 2005). A confirmatory factor analysis revealed acceptable fit statistics: *χ²*(2) = 330.37, *p* = .00, *CFI* = 1.00, TLI = .98, *RMSEA* = .17.

#### Smoking-Specific Rules

As previous research mainly focused on house rules with respect to smoking, new indicators for smoking-specific rules were developed. These indicators included: (1) I am allowed to try out smoking a cigarette, (2) I am allowed to smoke now and then, (3) I am allowed to smoke regularly. Answer categories ranged from ‘definitely not true’ to ‘definitely true.’ A confirmatory factor analysis revealed acceptable fit statistics: *χ²*(1) = 274.52, *p* = .00, *CFI* = 1.00, TLI = .99, *RMSEA* = .22.

### Covariates

All analyses controlled for gender (boy vs. girl), age (ranging from 12 to 16), and educational level (low vs. high). Educational level, which is a strong predictor of adolescents’ future socioeconomic status (ROA 2009), was included as a dummy variable. While the Dutch educational system consists of four educational levels, many secondary schools are specialized in teaching either pre-vocational training and lower academic education or medium and higher academic education. Therefore, pre-vocational training and lower academic education were combined (i.e., low educational level) and medium and higher academic education were combined (i.e., high educational level) for the purpose of the current analyses.

## Statistical Analyses

First, we provide descriptive statistics to identify the prevalence of adolescent risk behaviors and the amount of parental support, control, and concrete rules on adolescent smoking and drinking behaviors for male and female adolescents, youth from the different age groups, and students with high and low educational levels.

Second, we designed a structural equation model in Mplus version 6.11 (Muthén and Muthén 1998–2010). In this model, adolescent risk behaviors were predicted by the four parenting practices. Because concrete parental rules on alcohol use and smoking are a way to assert parental control, these variables were included as mediators in the association between parental control and adolescent risk behaviors, thereby following the example of previous studies (e.g., van Zundert et al. 2006).

Third, three moderation analyses were performed to examine whether the effects found in the final model were equally present for (1) boys and girls, (2) adolescents from different age groups (early vs. mid adolescents), and (3) adolescents with a low versus high educational level. For each moderation analysis, the model fit of two models was compared: (1) a model in which all paths were freely estimated for the two groups (i.e., boys and girls, early and mid-adolescents, and students with a low and high educational level) and (2) a model in which the paths from the parenting practices to the risk behaviors were constrained to be equal across groups. The model fit comparison was based on two criteria, which both needed to be met in order to conclude that there was a significant difference between the models. These criteria were (1) the chi square difference test, a traditional test that indicates whether the fit of two models differs significantly but which is sensitive to sample size and (2) Chen’s criteria, which are not sensitive to sample size (i.e., models differ significantly in fit if *ΔCFI* > .01, *ΔTLI* > .01, or *ΔRMSEA* > .005) (Chen 2007). If the freely estimated model had a significantly better fit based on both criteria, then this model would be preferred. If not, then the most parsimonious model (i.e., the constrained) model would be preferred.

Data were weighted for educational level, grade, gender, and urbanicity with poststratification weights. All analyses were corrected for cluster effects of pupils within the same school (primary sampling unit). The range from missing values per variable ranged from 0 % to 3 % (early sexual intercourse). A robust weighted least squares estimator (WLSMV) was used in combination with full information maximum likelihood estimation to deal with missing data (Enders and Bandalos 2001). Because the *N* of our sample was large, we used significance criteria of α = .001 and α = .01.

## Results

### Descriptive Statistics

The prevalence rates of adolescent risk behaviors and parenting practices as reported by adolescents are presented in Table 1. No gender differences existed with respect to risk behavior and parenting practices, except for parental control; girls experienced more parental control than did boys. Furthermore, older adolescents experienced less parental control and concrete rules and engaged more in all risk behaviors compared to younger adolescents. Finally, youth with a low educational level experienced less parental support and control and engaged more often in smoking tobacco, early sexual intercourse, and binge drinking compared to youth with a high educational level.

**Table 1:** Prevalence of adolescent risk behavior and parenting practices stratified by adolescent age, gender, and educational level (%, *N* = 5,642)

	Total	Gender^1^		Age^1^					Educational level^1^	
		Boys	Girls	12	13	14	15	16	Low	High
Adolescent risk behavior										
Daily tobacco smoking	6.8	7.0	6.6	.1^a^	2.6^b^	5.2^b^	12.2^c^	19.2^c^	10.2^a^	3.0^b^
Binge drinking in the previous month	27.5	28.0	26.9	8.0^a^	14.2^a^	25.3^b^	44.8^c^	56.6^c^	32.3	22.0
Lifetime cannabis use	11.8	13.6	9.9	.5^a^	3.5^b^	10.6^c^	22.2^d^	28.9^d^	12.6	10.8
Early sexual intercourse	11.7	12.3	11.0	1.0^a^	3.3^a^	8.5^b^	20.6^c^	33.6^d^	14.9^a^	8.2^b^
Parenting practices										
Much parental support^2^	88.3	88.0	88.6	91.1	90.4	87.3	86.6	84.8	85.9^a^	91.0^b^
Much parental control^3^	35.4	26.4^a^	44.6^b^	46.0^a^	39.3^a^	31.3^b^	30.4^b^	28.1^b^	31.3^a^	40.0^b^
Strict parental rules on adolescent alcohol use^4^	38.2	39.7	36.6	65.4^a^	51.6^b^	34.7^c^	17.8^d^	14.0^e^	39.5	36.7
Strict parental rules on adolescent smoking^5^	70.6	70.2	71.0	85.8^a^	81.5^a^	68.4^b^	57.3^c^	53.7^d^	68.5	72.9

^1^In rows, values with different superscripts are statistically different from each other at *p* < .01 (separately for gender, age, and educational level)

^2^% scoring > 3.5 on the parental support scale (1–5)

^3^% scoring > 4.5 on the parental control scale (1–5)

^4^% scoring > 4.5 on the parental rules on adolescent alcohol use scale (1–5)

^5^% scoring > 4.5 on the parental rules on adolescent smoking scale (1–5)

### To What Extent Do Parenting Practices Predict Adolescent Risk Behaviors?

**Fig. 1:**
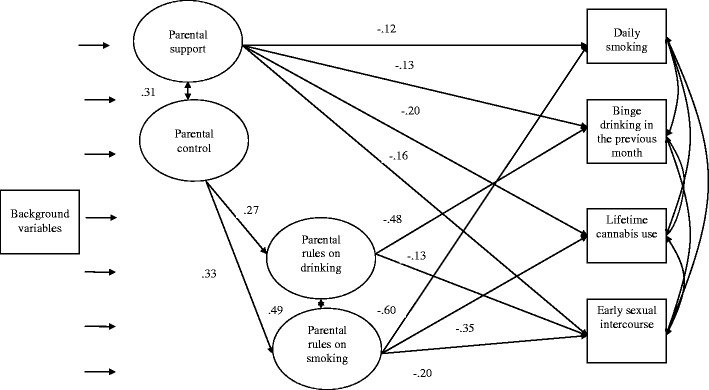
General and specific parenting practices predicting adolescent risk behavior. *Note.* Model fit statistics of this model: *χ²*(184) = 875.42, *p* = .000, *CFI* = .99, TLI = .99, *RMSEA* = .03. Indicators of the four latent constructs representing parenting practices were not presented for readability reasons; these can be found in the methods section. Information on factor loadings and residual error terms is available from the authors. All standardized estimates are statistically significant (*p*s < .001)

Our model, which aimed to predict adolescent risk behaviors by the four parenting practices, had a good fit: *χ²*(184) = 875.42, *p* = .000, *CFI* = .99, *TLI* = .99, *RMSEA* = .03. The model estimates of the final model are illustrated in Fig. 1.

Parental support was negatively associated with all four risk behaviors. There was no direct association between parental control and risk behavior; however, via the mediator parental rules on smoking, parental control had an indirect effect on adolescent daily smoking (*β* = -.20, *p* < .001), lifetime cannabis use (*β* = -.12, *p* < .001), and early sexual intercourse (*β* = -.07, *p* < .001). In addition, via the mediator parental rules on alcohol use, parental control had an indirect effect on adolescent binge drinking (*β* = -.13, *p* < .001) and early sexual intercourse (*β* = -.03, *p* < .01).

The direct associations between parental rules on smoking and drinking were especially high with the targeted behaviors: *β = -*.48 (alcohol rules and adolescent binge drinking) and *β =* -.60 (smoking rules and adolescent smoking behavior). Interestingly, the associations with other behaviors were also quite strong and ranged from *β* = -.13 (alcohol rules and adolescent early sexual intercourse) to *β* = -.35 (smoking rules and adolescent cannabis use).

Furthermore, parental support and parental control were strongly correlated, as were parental rules on adolescent smoking and drinking. The four risk behaviors were all correlated and ranged from *r* = .31 (binge drinking and early sexual intercourse) to *r* = .45 (smoking and cannabis use) (*p*s < .001).

In total, this model explained 47.3 % of the variance in binge drinking, 61.5 % of the variance in daily tobacco smoking, 44.0 % of the variance in lifetime cannabis use, and 39.4 % of the variance in early sexual intercourse.

### Moderation by Adolescent Age, Gender, and Educational Level

To test whether the results of our model applied similarly to different subgroups of youth, three moderation analyses were conducted. For each analysis, the model fit of the model in which all regression paths were constrained across the groups, was compared with the freely estimated model. In the analysis that compared early versus mid adolescents, both the chi square difference test (Δ*χ²* (16) = 22.09, *p* = .14) and Chen’s criteria (*ΔCFI* = .001, *ΔTLI* = .001, *ΔRMSEA* = .002) showed that the fit of the freely estimated model (*χ²*(400) = 1148.97, *p* < .001, *CFI* = .99, *TLI* = .99, *RMSEA* = .03) was not significantly better than the fit of the constrained model (*χ²*(416) = 1106.70, *p* < .001, *CFI* = .99, *TLI* = .99, *RMSEA* = .02), which indicates that the associations between parenting practices and adolescent risk behaviors were similar for early and mid adolescents.

In the gender analysis, the chi square difference test (Δ*χ²* (16) = 21.37, *p* = .16) indicated that the freely estimated model had a better fit; however, Chen’s criteria (*ΔCFI* = .000, *ΔTLI* = .001, *ΔRMSEA* = .001) did not support this finding. The fit of both models was good: *χ²*(400) = 1194.10, *p* < .001, *CFI* = .99, *TLI* = .99, *RMSEA* = .03 for the freely estimated model and *χ²*(416) = 1189.12, *p* < .001, *CFI* = .99, *TLI* = .99, *RMSEA* = .03 for the constrained model. Based on the fact that the chi square test is sensitive to sample size and, on the observation that the more robust fit indices were hardly different (Chen’s criteria), we concluded that the constrained model was preferred, which indicates that the associations in our model did not differ for boys and girls.

Finally, in the analysis that compared adolescents with a low and high educational level, the model fit of the constrained (*χ²*(416) = 983.83, *p* < .001, *CFI* = 1.00, *TLI* = .99, *RMSEA* = .02) and freely estimated model (*χ²*(400) = 1023.57, *p* < .001, *CFI* = .99, *TLI* = .99, *RMSEA* = .03) did not significantly differ based on the chi square difference test (Δ*χ²* (16) = 18.64, *p* = .29) or Chen’s criteria (*ΔCFI* = .001, *ΔTLI* = .000, *ΔRMSEA* = .002), which indicates that the associations were also similar for students with a low and high educational level. In conclusion, the associations in our model were similar in strength for all subgroups of youth.

## Discussion

The present study yielded four main findings. First, it shows that both parental support and control (via concrete parental rules) were associated negatively with adolescent smoking, drinking, cannabis use, and early sexual activity. Second, concrete parental rules on smoking were associated with a lower likelihood of adolescent smoking and concrete parental rules on alcohol use were associated with a lower likelihood of adolescent drinking. These effects were stronger than the effects of parental support and control. Third, parental rules also had an effect on other risk behaviors: rules on alcohol use predicted a lower likelihood of early sexual intercourse and rules on smoking predicted a lower likelihood of adolescent cannabis use and early sexual intercourse. Finally, the abovementioned associations were equally strong for early and mid adolescents, boys and girls, and students with low and high educational levels.

The first finding, that adolescent smoking, drinking, cannabis use, and sexual activity were negatively influenced by parental support and control, confirmed our hypothesis and is consistent with previous research (Castrucci and Gerlach 2006; Chen et al. 2005; de Graaf et al. 2010, 2011; Harakeh et al. 2010; Roche et al. 2008; Ryan et al. 2010; van Zundert et al. 2006). As general and specific parenting practices were both present in our model, it was possible to identify the relative strength of associations of both types of practices. Compared to parental support and control, concrete parental rules that target specific risk behaviors clearly had a stronger association with (1) the targeted behaviors (i.e., smoking and drinking) and (2) related risk behaviors that were not directly targeted (i.e., cannabis use and early sexual intercourse). This finding may be explained by concrete rules being conceptually closer to the risk behaviors compared to parental support and control. It is important to stress, however, that parental support and control (via the mediation of rules) both had significant associations with all risk behaviors.

In accordance with previous studies, we found that concrete rules that target adolescent alcohol use were related to a lower likelihood of adolescent drinking (e.g., van Zundert et al. 2006). Interestingly, while previous studies have found modest or contradictory effects of parental rules on smoking, the present study revealed a strong association with adolescent smoking behavior. This difference in outcome is likely to be caused by the fact that we used a different definition of smoking rules than did most previous studies. Specifically, previous studies have focused on general house rules (Andersen et al. 2004; de Leeuw et al. 2010; Henriksen and Jackson 1998; den Exter Blokland et al. 2006; Harakeh et al. 2005) or rules which were targeted specifically at the adolescent, but which were still limited to the home context (Huver et al. 2006; Pennanen et al. 2012), while parental smoking rules in the current study were defined as the adolescent being allowed to smoke by his or her parents in general (i.e., not restricted to the home). Rules aimed directly at the behavior of the adolescent, independent of context, can be expected to be more relevant than are general rules about smoking at home.

Our finding that concrete parental rules that target adolescent smoking and drinking behaviors are also related to other types of risk behaviors is innovative and may be explained by the fact that the four risk behaviors under study are closely interrelated and often occur in a similar context. For example, alcohol use and sexual intercourse are both related to a context of going to bars and pubs at night. If youth are allowed to drink alcohol, then they are more likely to find themselves in such a context; therefore, they are not only more likely to drink alcohol, but also to meet peers, date, and have sexual intercourse. A different explanation could be that adolescents experience parental rules on smoking and drinking in a broader context; they expect their parents to be consistent (i.e., if they are not allowed to smoke, they are definitely not allowed to use cannabis). A final explanation is that parents who set rules with respect to adolescents’ smoking and drinking behaviors, generally tend to monitor their children more so compared to parents who do not set such rules. This would explain why, for example, parental rules on smoking and adolescent delay in sexual debut are associated, even though they are not very close conceptually.

The finding that the parenting practices under study were related to a reduction in adolescent risk behaviors similarly for early and mid adolescents, boys and girls, and youth with different educational levels, was somewhat surprising. Specifically, the findings show that, in subgroups of youth who experience less parental support, control, and concrete rules (e.g., boys, mid adolescents, adolescents with a low educational level), the effects of these parenting practices are similar. Similarly, for subgroups of youth who may spend less time with their parents and more time with peers (most notably older youth and youth from lower educational levels; Currie et al. 2008; De Looze et al. 2012), parental rule setting may be a powerful practice to prevent youth from engagement in substance use. The results of the present study underline that parental influence remains of major importance, also when youth begin to spend less time at home and experience less physical influence, support or control from their parents.

## Strengths and Limitations

The present study has several strengths. First, our analyses were based on a national representative sample of adolescents; therefore, our conclusions can be generalized to the entire adolescent population (aged 12 to 16) in the Netherlands. Second, our model explained a relatively large part of the variance in adolescent risk behavior, which indicates that our predictors (i.e., parenting practices) contribute substantially to explaining adolescent engagement in risk behaviors. Finally, this study does not only have implications for academia, but also for the public health domain. It is our hope that the current results can guide prevention workers toward the development of effective and efficient prevention programs.

Our study also has a few limitations. First, it was based on cross-sectional data; therefore, we cannot make any causal inferences. Based on our findings, it is not clear whether parenting practices influence adolescent behaviors, whether adolescent behaviors influence parenting practices, or whether there is a third variable that influences both parenting practices and adolescent risk behaviors. Factors that may impact both parental support, control, and rule setting and adolescents’ propensity for risk behavior include genetics (McGue et al. 2000; Pagan et al. 2006; Rose and Kaprio 2008), parental substance use (Barnes et al. 2000; Koopmans and Boomsma 1996; Latendresse et al. 2008), parental attitudes on substance use (Denton and Kampfe 1994), and familial mental health concerns (Repetti et al. 2002). As intervention studies have convincingly demonstrated an effect of parenting practices on adolescent risk behaviors (e.g., Koning et al. 2011b), it is likely that our findings (at least partly) reflect an effect of parenting practices on adolescent substance use and early sexual debut. Yet, future intervention research is needed to clarify these mechanisms in more detail.

A second limitation is that our data were based on adolescent self-report, which entails the risk of socially desirable answers. To counter this potential bias, anonymity was stressed by interviewers before youth completed the questionnaires. Furthermore, it is important to stress that we only used adolescent reports of parenting practices, not parental perceptions.

Finally, the present study focused on parental rules with respect to adolescent smoking and drinking behaviors; however, no rules on cannabis use and early sexual intercourse were included. Of note, such rules have rarely been the subject of investigation, probably because they are generally implicit. Yet, parents who set boundaries regarding alcohol use and smoking may be likely to set rules about other risk behaviors, such as cannabis use, as well. Unfortunately, for the present study, data on such rules were not available. Future research may investigate the effects of cannabis-specific parenting practices on adolescent cannabis use. Additionally, the association between parenting practices regarding adolescent sexual behaviors and adolescent sexual activity may be investigated in more detail. As many parents find it hard to discuss sexual behaviors with their children (de Graaf et al. 2005; van Dorsselaer et al. 2010), they are more likely to set indirect instead of explicit rules with respect to adolescent sexual behaviors. For example, parents may set rules with respect to going out at night, sleeping over at friends’ houses, and the use of internet. For example, to protect their children from unwanted sexual solicitation or harassment, parents may set the rule that they should never react to sexual solicitations by strangers or people they do not know very well (Ybarra and Mitchell 2008).

## Implications

The findings of the present study have implications for the public health domain. Our results indicate that the four risk behaviors under study are related to parental behavior in similar ways. This suggests that a broad intervention program focusing on a limited number of parenting practices (i.e., general as well as specific) may be effective in simultaneously reducing adolescent substance use and early sexual intercourse among 12 to 16-year-old youth. Such broad programs would be more cost efficient and probably have higher rates of parental compliance compared to the implementation of separate programs that target a single risk behavior.

It is important to note that an intervention study is necessary to actually conclude that such a program would be beneficial. Currently unknown factors may play a role in causing both parents to adopt certain parenting practices and adolescents to engage less in risk behaviors. Additionally, based on the current data, we do not know how parents who do not engage in certain practices would react if they were encouraged to adopt these practices. If an intervention study succeeds in demonstrating that parenting practices can be modified and that this has a desired effect on adolescents, only then could such a program be deemed as evidence-based and be implemented. Therefore, the current study should be perceived as a modest step toward exploring the possibility of designing broader intervention programs that reduce adolescent smoking, drinking, drug use, and early sexual intercourse.
